# A Sensor Network Approach for Violence Detection in Smart Cities Using Deep Learning

**DOI:** 10.3390/s19071676

**Published:** 2019-04-08

**Authors:** Marius Baba, Vasile Gui, Cosmin Cernazanu, Dan Pescaru

**Affiliations:** 1 Computers and Information Technology Department, Politehnica University of Timisoara, Timisoara 300223, Romania; mariusb007@yahoo.com (M.B.), cosmin.cernazanu@cs.upt.ro (C.C.); 2 Everseen Limited, 4th Floor, The Atrium, Blackpool, T23-T2VY Cork, Ireland; vasile.gui@everseen.com

**Keywords:** sensor networks, deep learning, action classification, violence detection, smart cities

## Abstract

Citizen safety in modern urban environments is an important aspect of life quality. Implementation of a smart city approach to video surveillance depends heavily on the capability of gathering and processing huge amounts of live urban data. Analyzing data from high bandwidth surveillance video streams provided by large size distributed sensor networks is particularly challenging. We propose here an efficient method for automatic violent behavior detection designed for video sensor networks. Known solutions to real-time violence detection are not suitable for implementation in a resource-constrained environment due to the high processing power requirements. Our algorithm achieves real-time processing on a Raspberry PI-embedded architecture. To ensure separation of temporal and spatial information processing we employ a computationally effective cascaded approach. It consists of a deep neural network followed by a time domain classifier. In contrast with current approaches, the deep neural network input is fed exclusively with motion vector features extracted directly from the MPEG encoded video stream. As proven by results, we achieve state-of-the-art performance, while running on a low computational resources embedded architecture.

## 1. Introduction

The Smart City approach is considered as a promising solution to the problems related to enhanced urbanization [1]. Its implementation depends on the capability of gathering and analyzing huge amounts of various live urban data. They are collected mainly from public and private sensors networks run by various agencies or private bodies. Among other data types, video streams especially provide valuable information collected directly from the street. Smart city surveillance covers a large spectrum of applications, which include, among others, urban traffic monitoring systems [2], building structural damage detection [3], violence detection [4], and disasters management [5]. Human operators might be easily overwhelmed with the number of video streams. Therefore, an important research effort was directed to developing methods to process automatically such information in order to monitor abnormal behavior, and to discard safely irrelevant information.

Violence detection represents an important issue in smart city surveillance. A cost-effective solution uses a wireless sensor network-based infrastructure. However, such a solution implies nodes with low computational power and limited communication bandwidth. Various approaches were proposed to overcome these problems. The authors of [6] reduce the processing demands by using only the audio data. Other methods, as the one proposed in [7], implies high bandwidth for video streams transfer to a powerful base station.

In contrast with existing approaches, the work reported here proposes an algorithm running at the sensor node level able to detect the violence from camera input, and to reduce the communication load by transferring only alerts. State of the art algorithms for violence detection are based on computationally intensive computer vision techniques like segmentation, tracking, and action recognition [8]. Violence detection is considered a particular case of human action recognition [9]. Complex background, scale, and occlusion make activity recognition very difficult. Moreover, violence detection poses also specific issues that start with a proper definition of violence. Discrimination between violent and non-violent scenes is sometimes difficult even for humans. Some activities like running, dancing or person to person interactions are very similar in appearance to violent behavior.

Due to the size of video data collected from a large cameras network, the required computational power is significant. Therefore, the algorithms based on handcrafted features using image and video processing do not represent the best solution. Deep Neural Networks (DNNs) and Deep Learning (DL) approaches [10] are more reliable for handling such large-scale data. A convolutional DNN combines in various ways convolutional layers, pooling layers and fully connected layers to automatically extract features from images and to use these features for predictions. Such a network can be trained by using a backpropagation algorithm based on a loss function. Convolutional DNNs were inspired by biological processes (organization of the animal visual cortex) and were mainly created for image processing and analysis. Encoding certain properties into the architecture results into fewer parameters and easier training.

Due the recent investments in specialized highly parallel hardware as Graphical Processing Units (GPUs) and Tensor Processing Units (TPUs) [11], the deep learning is expected to have more and more impact in almost all industries. DNNs can be used to implement real-time algorithms for object detection, object tracking, face recognition, image classification, and action recognition. However, an important challenge for deep learning solutions is the deployment on smaller and widespread processing systems. 

Sensor network video surveillance in urban area involves large amounts of small nodes capable of video sensing. A possible approach is based on a powerful central node able to implement DL solutions by processing video streams gathered from the network nodes. This centralized approach has a major drawback related with the bandwidth requirement considering high resolution video streaming. A better solution involves distributed processing at the level of each node. The main issue in this case is the low computational power available for implementing DL algorithm.

To address the problem of implementing a DL algorithm on a low resource node we consider a Raspberry PI architecture equipped with a USB camera. On this architecture we implement a six layers deep neural network able to classify video sequences as violent or non-violent. In contrast with previous work, we use as DNN input exclusively motion feature vectors extracted from the Motion Picture Expert Group (MPEG) encoded video stream. Since the vectors encode motion for 16x16 image blocks, we obtain 256-fold network size reduction. We train the DNN to recognize frames corresponding to violent behavior. To cope with temporal characteristics of fight scenes, we feed the output of the DNN to a temporal domain filter. The cascaded architecture of the system is computationally effective and, as we prove, obtains state of the art performance.

The rest of the paper is organized as follows: Section 2 presents some related work used as the starting point for our research. Section 3 presents in details the proposed method while the next Section discusses some optimization work. The method validation is presented in Section 5, and the last Section concludes the work. 

## 2. Related Works

Violence detection in video is primarily needed to enhance citizen security, to prevent children from watching movies containing violent behavior, or to enable content-based video search. Violent activities can be recognized by analyzing human actions. We are interested in actions leading to suspicion about violence in sensitive urban areas. Work in violence detection draw inspiration from the larger field of activity recognition. Approaches to both violence detection and action recognition fall into two categories: traditional, also called handcrafted approaches, and deep learning approaches. A comprehensive view on action recognition, including violence detection, using both handcrafted approaches and deep learning is presented in [12].

Among the oldest attempts to detect violence in video is the work of Nam [13]. The authors used a combination of flame and blood detection in conjunction with sound and motion features. Blood and motion were also used for violence detection in [14] along with skin detection features. More recently Chen [15] combined motion features with face and blood detection. Audio and motion feature statistics have been fed to a k-Nearest Neighbor classifier in [16] in a data fusion framework. While blood, explosions and specific fight-related sounds may be useful for violence detection in movies, they can be rarely found in urban violence scenarios. 

The most powerful information needed to recognize and characterize human physical actions and interactions is motion. Such activities usually involve specific motion patterns, and they take place in relatively low time intervals. Early work on activity analysis in video concentrates on holistic representations of motion, like motion history or motion energy images [17]. A more recent trend is to use localized representations. Interest points like the Space-time Interest Points (STIP) or Space Time Cuboids [18] can help to characterize image motion. The information provided by these features is further refined by generating higher level features like motion trajectories. Related to STIP is the Motion Scale Invariant Feature Transform (MoSIFT) introduced by Chen [19], which encodes local appearance and motion. Both are generalizations of Loeve’s Scale Invariant Feature Transform (SIFT) [20]. Their features have been used with similar performance for violence detection by Neivas [4]. Interest points are typically used with the popular Bag of Words framework [21,22], followed by a Support Vector Machine (SVM) classifier [23]. Motivated by Weber’s low of perception, Zang [9] proposed the Motion Weber Local Descriptor (MoWLD). Like MoSIFT, the descriptor captures both local motion as histogram of optic flow and appearance as histogram of Webber local descriptor. 

Motion trajectories synthetize well the motion information over a set of frames. A representative work using motion trajectories for analyzing activities in a parking lot is reported in [24]. Trajectories are generated by a combination of methods involving foreground/background segmentation [25] and a multi-hypothesis tracker [26]. Inference is obtained via random forest and support vector machine classifiers. Dense trajectories described in [27,28] are used in [29] to capture both shape and motion features to detect urban fight.

One of the most powerful features in motion analysis is the optical flow. Optical flow research has a long history [30]. A popular method used in this work as a reference method is the Farneback optical flow algorithm [31]. Although computationally expensive, optical flow is widely used in violence detection. For example, multiscale optical flow features are incorporated in the MoSIFT and MoWLD feature vectors discussed earlier. Hassner [32] proposed the violent flows descriptor (ViF) for crowd violence detection. While the ViF can be computed efficiently and works well in the crowd scenario, it is less effective on non-crowd video data. To alleviate the problem, Gao [33] proposed the Oriented Violent Flows (OViF) which is a new version of the ViF. Unfortunately, it performs poorly in crowd violence detection. Recent approaches to optical flow computing are based on DNN and prove significant performance improvement [34,35]. These solutions also involve lot of computational resources. For example, the Liteflownet model proposed in [35] requires over 160M parameters. 

Currently DNNs also challenge state of the art in activity recognition. Frequently used architectures in activity recognition are spatio-temporal convolutional networks [36], and recurrent networks [37,38]. An extensive survey on activity recognition mainly focused on DNN solutions can be found in [39]. 

A major advantage of using DNNs consists in the ability of such networks to automatically discover features adapted to the targeted problem. However, there are some issues in this approach implementation. An important one is the dependency between the performance and the dimension of the data set used for training. Knowledge transfer can be used to alleviate this overfitting problem. It involves pretraining the network on a larger database, and more generic in content, followed by fine tuning on the targeted data. Another issue is the significant demand of hardware resources, especially in case of real time applications. All of them are critical in achieving the goal of the work presented here. 

One possible approach to capture the time domain evolution of activities is to feed the DNN with a set of consecutive frames. The simplest architecture using this idea, called early fusion is using only one spatio-temporal volume of the video stream which is slide in the time domain [40]. An alternative solution, known as late fusion, is to input several single frames to several CNNs, which are fused at the final fully connected stage [40]. The authors report here some experiments with an intermediate architecture called slow fusion. It involves several CNNs fed with overlapping chunks of input frames. The CNNs are gradually fused in a multi-resolution manner toward the final, fully connected layer. Slow fusion needs less parameters to be learned and outperforms the other version of 3D approach.

More complex solutions for action recognition involves DNNs composed of Long Short-term Memory (LSTM) networks and 3D CNNs [41]. This approach uses descriptors generated by the CNN to feed the LSTM network. Memory feature enables the LSTM to learn action sequences. However, training such LSTMs is a very challenging task, and in our case, at runtime, overcomes the available hardware resources.

Despite being widely used in activity recognition, in the field of violence detection, DNN approaches are still scarce. Some attempts are presented in [42,43,44]. Xu [42] proposes the Appearance and Motion DeepNet (AMDN) to learn discriminative feature representations for anomalous event detection. However, the actual classification is performed by an SVM classifier. Dai et al. [43] use a data fusion strategy, combining a dual stream convolutional neural network with a LSTM network and several conventional features, like improved dense trajectories features according to [28], trajectories-based features, including histograms of oriented gradients, histograms of optical flow, motion boundary histograms and trajectory shape descriptors. To perform dimensionality reduction, the authors use Fisher vector encoding. Again, the SVM classifier makes the final decision. Sudhakaran [44] uses a convolutional LSTM instead of a fully connected LSTM. By doing so, the model becomes capable of encoding spatio-temporal information in its memory cell and reduces overfitting. In addition to RGB and optical flow input modalities, Zhou [45] uses the image acceleration field to train their FightNet model. The image acceleration field is computed from two consecutive frames of the optical flow. 

## 3. A Solution for Action Recognition in Low Hardware Resources Environment

### 3.1. General Description

We propose here a distributed surveillance solution for violence detection designed for a typical sensor network environment. The network consists in a set of low complexity processing nodes and a powerful central station. Nodes are responsible to obtain a high recall in detecting violent events. The aim is to avoid missing such events and to obtain high accuracy to minimize the false alarms. Video sequences classified as violent actions are sent to the central unit for further processing and action triggering. 

One distinctive feature of the proposed approach is that we entirely rely on motion features, by completely disregarding appearance information. The decision is based on results from previous work on human activity recognition [46], which demonstrated that motion is far more informative than appearance information. Furthermore, to make effective use of appearance information in classification, given the often-subtle differences between the appearance of a person running or kicking in a fight, one needs huge amounts of data and complex representation models. An additional advantage of relying exclusively on motion information is a high invariance to illumination changes. This includes safe operation in night vision scenarios, implying infrared monochromatic sensing. 

The second major decision is to represent motion with MPEG flow motion vectors, instead of the more widely used optical flow. The comparative study presented in [47] demonstrates the effectiveness of this approach in action recognition. Results are asymptotically identical and sometimes better compared with optical flow-based methods. The optical flow feature extraction is known to be very expensive from the computational point of view. On the contrary, MPEG flow vectors can be directly extracted from the MPEG encoded video stream, with no additional processing. Since each flow vector encodes the motion of a 16×16 image block, a significant dimensionality reduction of the input size for the classification CNN is obtained. While using handcrafted features to represent the input, the CNN still has to discover new features from the motion vectors. In this way it will capture relevant information regarding the spatio-temporal distribution of the motion vectors. 

The third major design decision was to put spatial and temporal information processing in a cascaded architecture, which again brings considerable simplification of the model, processing and optimization tasks. Indeed, considering a set of *T* frames of size *N* × *N,* the processing complexity is reduced from *N* × *N* × *T* to (*N* × *N + T*). Furthermore, the temporal processing unit itself has a cascaded architecture.

### 3.2. The Video Processing Unit

The capability of DNNs to automatically discover optimized features may be very tempting for replacing the skilled work and insight in the application specificities, needed to generate handcrafted solution, but there is a price to obtain this performance. The skilled work should be substituted by the rather repetitive task of data labeling. Often this task can benefit from a sum of large labeled datasets for DNN training already built and made publicly available in many domains. Unfortunately, when it comes to develop applications in new domains this advantage is most often lost. 

Labeled databases containing violence scenarios in surveillance video are scarce. We used here two important surveillance video databases publicly available online as BEHAVE [48] and ARENA [49]. As they contain a relatively small set of violent sequences, we decide to use both in this work.

The solution presented here aims to combine the power of DNNs with the efficiency of some well-chosen handcrafted motion features. To enable the system to learn from the relatively small available datasets, we feed the network with a reduced set of motion features, namely MPEG flow vectors. These vectors express the motion of 16 × 16 image blocks. The spatio-temporal distribution of these low-level features is capable of capturing and discriminating a large variety of motion types, as walking, running or jumping. To automatically discover higher level features from MPEG flow data, needed to discriminate patterns of motion corresponding to violent versus non-violent behaviors, we use a combination of a CNN and temporal domain filter. The architecture of this filter is detailed in a further section.

**Figure 1:**
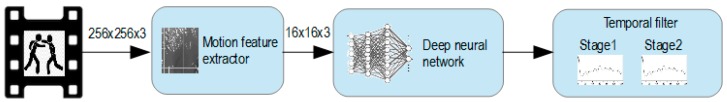
The main steps of the algorithm running on a sensor node.

To speed-up the processing, we use in our approach a reduced number of convolutional layers. As we prove in the validation section, we can preserve the detection performance even in conditions of using a relatively small number of training samples. Therefore, we achieved to design a real time distributed system able to detect violence in urban areas. It is based on Raspberry PI nodes running the algorithm depicted in Figure 1.

### 3.3. Low Level Feature Extraction

We extract the MPEG flow [47] motion features and use them as the input of our DNN. These features represent estimators of 16x16 image micro-block motions which are computed and are used by the image encoder to compress the video data. The usefulness of these features in action recognition has already been demonstrated in [46,47]. In [46] the MPEG flow features are used in the bag of words approach, while in [47] the authors use spatio­temporal CNN.

**Figure 2:**
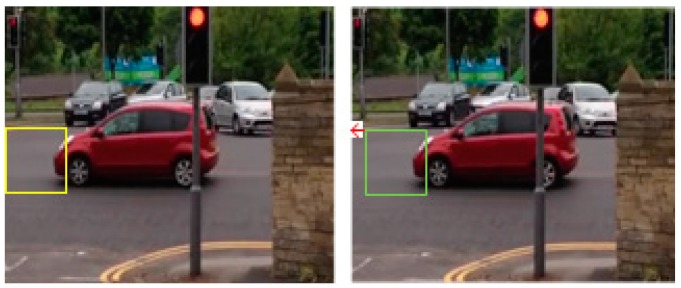
The principle of block motion estimation. Left image: block in current frame marked with yellow rectangle. Right image: best match block in previous frame, marked with green and motion vector marked with red arrow.

The motion of all pixels in a 16 × 16 micro-block is approximated by a unique motion vector. This vector is computed by searching in the previous frame a best match for the currently encoded micro-block, as illustrated in Figure 2. One currently encoded micro-block is represented in the left image by a yellow rectangle, while its corresponding best match in the previous frame is represented in the right image by a green rectangle. The red arrow in the right image is the motion vector of the micro-block. The encoder represents this vector by specifying the reference positions for the currently encoded micro-block and of its corresponding image block in the previous frame as presented in (1).

(1) $$v_{m}=p_{source}\;-\;p_{destination\;}$$

To find the best match, the encoder uses a distortion measure between the matched blocks, namely the sum of absolute differences *SAD*(**v**):

(2) $$S\mathrm{AD}\left(v\right)=\;\underset{p}{\sum }D\left(p,v\right)$$

In this equation, **p** represents the position vector of a current pixel in the encoded block and **v** is its estimated motion vector. The distortion measure at the pixel **p** is defined by Equation (3).

(3) D(p,v)=|Img(p)−Ref(p+v)|

The Img(p) is a pixel from the currently encoded micro-block and Ref(p+v) is a pixel from a block of the same size from the previous frame. The distortion measured for the entire block is expressed by the following equation:

(4) $$S\mathrm{AD}\left(v\right)=\;\underset{p}{\sum }D\left(p,v\right)=\underset{p}{\sum }\left|Img\left(p\right)-Ref\left(p+v\right)\right|$$

The computational cost of the MPEG flow features is very low. The encoder provides for each frame a list of 16 × 16 block motions where motion is present. The list contains pairs of source and destination position vectors. After decoding the video stream, we use the list entries to extract the motion vectors. However, in order to capture only significant motion, we apply a threshold *minMotionTh* on each extracted motion vector v_*m*_. The resulting vector v_*mth*_ is set to zero if the original vector magnitude is too small:

(5) $$v_{mth}=\{\begin{array}{c} v_{m\;},\;\mathrm{if}\;\left|v_{m\;}\right|>minMotionTh\\ 0,\;otherwise \end{array}$$

The following sequence describes the main steps of the algorithm used to estimate the magnitude of the motion from the MPEG flow. To remove noise, we consider in our experiments the value 3 for the *minMotionTh* threshold. The algorithm uses also a *nCorrection* offset, which is necessary to translate the interval [-128, 127] of vector axes magnitude to [0, 255]. Therefore, its value is 128.

1:
**Extract the set ξ of motion vectors from the MPEG stream**
2:
**Create an empty array ζ to store the motion on *X* and *Y* axes**
3: 4:
**FOR each motion vector *V* in ξ**

**DO**
5:  **Get the source and the destination points of the motion vector *V***6:  **Compute motion magnitude of *V***7:  **IF magnitude of *V* > *minMontionTh* THEN**8:   **Compute the *x* motion corresponding to the *X* axis and add *nCorrection***9:   **Store *x* into ζ on corresponding plane**10:   **Compute the *y* motion corresponding to the *Y* axis and add *nCorrection***11: 12:   **Store *y* into ζ on corresponding plane** **END**

**Figure 3:**
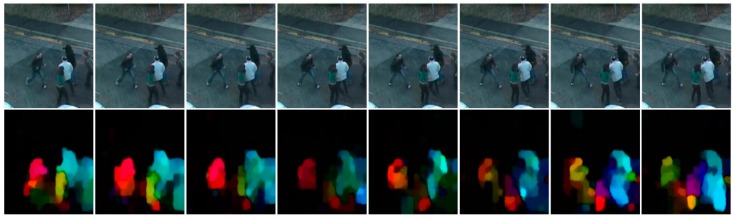
Optical flow on a violent sequence. First line contains eight frames from a fight sequence. The second line contains the corresponding color-coded MPEG flow feature maps (rescaled).

Figure 3 presents an example where the motion vectors are encoded into HSV for illustration purpose only. Here, the hue encoding the motion direction, and the value channel encoding the magnitude. The saturation channel was set to a constant. The RGB coordinates of motion vector image are fetched as input features to our CNN for convenience considering the expected input data format and range. To accommodate to the original image size, we show resized and interpolated RGB motion vector images.

### 3.4. The CNN Architecture

**Figure 4:**
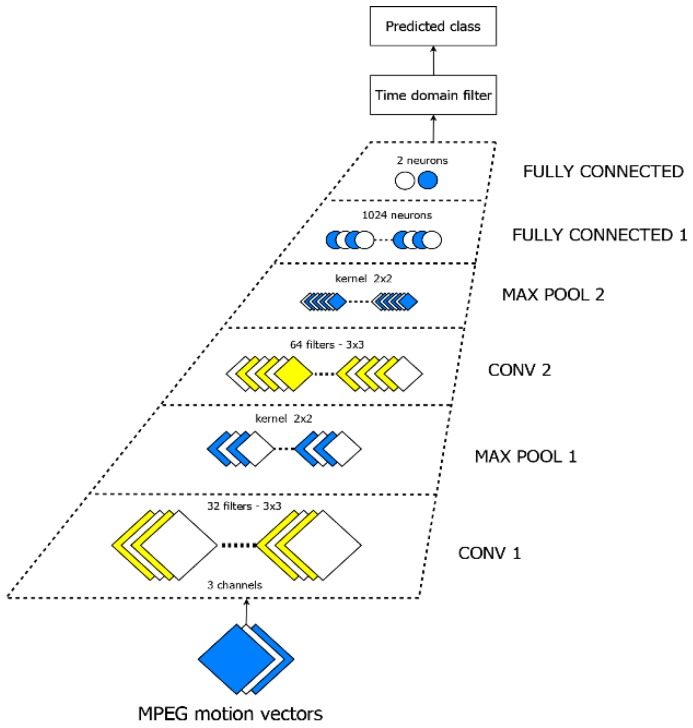
The classifier architecture.

The most important requirement for the CNN architecture is to be suitable for limited resources embedded processing. We considered here several options of well-known lightweight architectures like MobileNet [50] and SqueezeNet [51]. However, all of them are optimized for larger input size and have a quite large number of parameters. The smallest version of MobileNet has 0.47 M parameters and the SqueezeNet has 0.42 M after compression. Our architecture, shown in Figure 4, was inspired by the one proposed in [52] for the CIFAR dataset classification. It is a modified version of the one proposed by Krizhevsky [53] tailored for our problem and has only 21 K parameters. This choice is motivated also by another particularity. A more complex CNN suitable for a larger set of action recognition needs a larger dataset for training. The problem is approached in [46], which can recognize various human activities in videos captured either with static or dynamic cameras. To implement such a solution, important hardware resources and huge databases to learn the models are needed. 

None of the databases used by the authors of [46,54], and [55] are suitable for our application. Action categories as various sports, jogging or walking, are not annotated with respect to containing or not containing violence. Most of the clips are dynamic generated using cameras’ pan, zoom, and tilt functions. As a result, motion patterns occurring in different activities are far more complicated than their corresponding activities captured by static cameras. Classifying activities from databases require complex models, and consequently important processing power. This architecture has complexity of only 20,418 variables as weights and biases to be learned from the data.

The input is represented by a 16 × 16 × 3 matrix containing MPEG flow motion vectors. For a 256 × 256 pixels image the MPEG encoder provides 16 × 16 motion vectors, each corresponding to a 16 × 16 image block coded on three planes for implementation convenience. All convolution kernels are set to a 3 × 3 × 3 format in the space channel domain. The first stage contains 32 such filters. The second convolution layer extends the channel depth to 64 using the same convolution kernel. Each convolution layer is followed by a Rectified Linear Unit (ReLU) layer and a Max Pooling 2 × 2 layer. As a result, the first fully connected layer is fed with 64 activation maps of size 4 × 4. The last fully connected layer generates two outputs corresponding to the fight, and the non­fight class respectively.

### 3.5. Time Domain Filter

The output of the CNN is feed into a post processing cascade of two filters. They are used to capture the temporal evolution of the scene activity, and to give a more continuous output corresponding to human judgment and observer labeling.

The design of time domain filter is inspired by the fight action nature. It mimics the human judgments about fights. Usually, a fight action is composed of short intervals of motion. Few violent interactions, like kicking or punching, may be followed by a short pause with no motion. Wrestling is even more irregular with respect to motion intensity. To declare the observed action as violent, one must inspect a video for repeated motion and pause cues.

Finding a universal model able to classify actions from the point of view of the presence of violence is a challenging task. Here we use the output of a CNN, which has been optimized to classify frames based on MPEG flow. The output of the network in non-fight clips like running, group activity or with many people walking may exhibit sporadic decisions for violence. Conversely, within fight clips the output oscillates between decisions. To stabilize the output and to make it as similar as possible to human judgment, we propose a cascade of two temporal filters. The first one needs to fill in the gaps of still frames within fight frame sequences, while the second one needs to account for a minimum duration of a violent action.

We assume that the CNN output in clips with violent actions is frequently giving the violence class. Therefore, we propose to measure the frequency of fight labels within a time interval. A suitable tool to this purpose is the framework of probability density estimation. Since we cannot reasonably assume that the CNN output follows some known distribution, the use of a nonparametric density estimation is necessary. A nonparametric probability density estimator uses a continuous kernel *K* to estimate the density from a finite number of samples. The kernel was chosen to optimize the measurement of the density of violence class samples within a time window. The width of the time window defines the bandwidth of the estimator and acts as smoothing factor. The shape of the kernel is less critical. Therefore, we use a simple rectangular kernel expressed by:

(6) $$K_{h}\left(x\right)=\;\{\begin{array}{c} 1,\;\mathrm{if}\;\left|x\right|<h\;\\ 0,\;\mathrm{if}\;\left|x\right|\geq h \end{array}$$

where *x* represents the input variable and *h* is the scale parameter of the kernel. To count violence class samples within a window having the width 2*T* + 1 centered at time *t*_0_, we use the equation:

(7) $$n\left(t_{0};T\right)=\;\underset{t}{\sum }x\left(t\right)K_{T}\left(t-\;t_{0}\right)$$

In this equation *x*(*t*) is the output of the CNN, in our case a binary variable. It takes the value 1 for the violence class and 0 for non-violence. Note that the class probabilities predicted by the CNN can be used as well. The filter output is defined as:

(8) $$y\left(t\;;T,p\right)=\;\{\begin{array}{c} 1\;,\;if\;n\left(t;T\right)\geq p\left(2T+1\right)\;\\ 0\;,\;otherwise\; \end{array}$$

where *p* is the percentage of violent class samples within a kernel window of length 2*T* + 1.

To optimize the first filter, we need to find the best combination of values for parameters *p* and *T*. The second filter has a similar definition. However, its role is slightly different and so are its optimization criteria, as detailed in the next section.

## 4. Optimization

### 4.1. Data Preparation

To our best knowledge the only publicly available annotated databases generated with static surveillance cameras which are containing fight or violence activities are BEHAVE [48], ARENA [49] and UCSD [56]. These databases are not very extensive but still good-enough to train smaller models, as we need. The videoclips from the BEHAVE and ARENA contain a mix of violent and non-violent scenes used as positive and negative examples in our experiments. Since the UCSD database contains only non-violent scenes we choose not to use it in our work, and therefore not to affect the balance of the data. Moreover, from the first two databases we use for training purpose only the BEHAVE since the other contains only a few violent frames.

To maintain a balanced dataset with violent and non-violent activities, from the BEHAVE dataset we extracted 22 labeled video clips containing a total of 11,872 frames with the following types of activities:

- *Attack* (seven video clips)
- *Group*  (four video clips)
- *Run*  (six video clips)
- *Walk*  (five video clips)

We grouped the clips into two classes. The *Violence* class includes the *Attack* video clips, while the *Normal* class contains *Group*, *Run* and *Walk* video clips. The ARENA database contains only two video clips with violent behavior. Therefore, we decided to use it only for testing purposes. 

**Figure 5:**
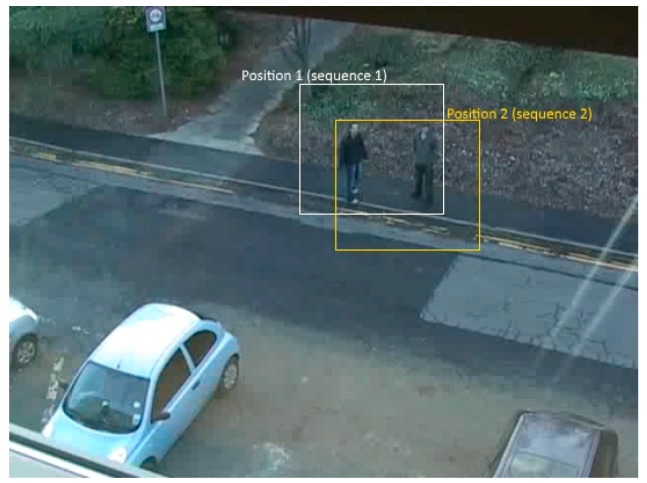
Example of augmentation ROIs.

To overcome the problem of the small amount of available video clips containing fights in the BEHAVE dataset, we chose to extend the dimension of the dataset using augmentation techniques. We prefer augmentation to alleviate the need for regularization, as this technique reduces the effective capacity of the classification model. We used the sliding bounding box technique to generate additional frames and clips, and to help the network learning translation invariance. A sum of Region of Interests (ROIs) with fixed sizes of 256 × 256 and variable positions were defined. These ROIs are positioned in the frame in such a way as to capture scene actors implied in fight. We illustrate this on Figure 5, where only the portions of a frame covered by the two ROIs are used for training or classification. To cover all regions of a higher resolution video, multiple 256 × 256 ROIs are needed to be used. By pseudo-randomly varying the position of a ROI, it is possible to capture the same fight with another spatial position in the active window. Therefore, from one action it is possible to generate many fight action samples, with the only constraint of keeping the actors in a ROI.

The second type of augmentation we used is horizontal image mirroring. This operation consists of flipping the image and changing the MPEG vectors accordingly. The first augmentation technique improves the overall accuracy by 6.78%. Using both shifting and mirroring brings an improvement of accuracy of 7.83%.

### 4.2. Learning the CNN Model

In order to fully exploit the available data, we used cross validation. To this purpose, we defined seven subsets of training and testing video clips. Each training subset contains six *Attack* video clips, and ten non-violent clips. The remaining *Attack* video clip and five non-violent clips were used for testing.

We trained the network using the Tensorflow GPU framework on an Intel I5, 4 GB RAM, 1 TB, NVidia GFORCE 1070ti PC. We used stochastic gradient descent search, with 50000 mini batches of 300 frames randomly selected from both classes. The Adam optimizer [57] was used to this end, with an initial learning rate of 0.0001 and default values for the other parameters [52] as *beta1* = 0.9, *beta2* = 0.999 and *epsilon* = 10^−8^. The Softmax cross entropy with logits was selected as the loss function. As regularization method we used dropout with keep probability set to 0.5.

### 4.3. Time Domain Filter Optimization

The goal of the system is to discriminate the violent sequences in a video stream considering a certain timeframe. The accuracy of this procedure is affected by the length of the examined time intervals. This length depends on the parameters *T* and *p* of the time domain filter.

The parameter *T* represents the length of the pre and post-frame examination intervals. It was set to the value of 10 to cover a time window of about one second. One second is a reasonable time for an observer to decide whether an activity is a fight. Longer time windows may be useful for lower false alarm rates. However, in the case of short fight sequences this choice would come with a lower detection rate. 

To optimize the percentile parameter *p*, we consider both criteria mentioned. The parameter *p* was selected at the level which is optimizing the *F*1 score of our system:

(9) $$F1=2\;\frac{precision*recall}{precision+recall}$$

Precision defines the percentage of true positive detections within the set of positive predictions, expressed as:

(10) $$precision=\;\frac{TP}{TP+FP}$$

where TP and FP stand for true positive and respectively false positive predictions. Recall defines the percentage of true positive predictions from all the positive events:

(11) $$recall=\;\frac{TP}{TP+FN}$$

where FN stands for false negative predictions.

**Figure 6:**
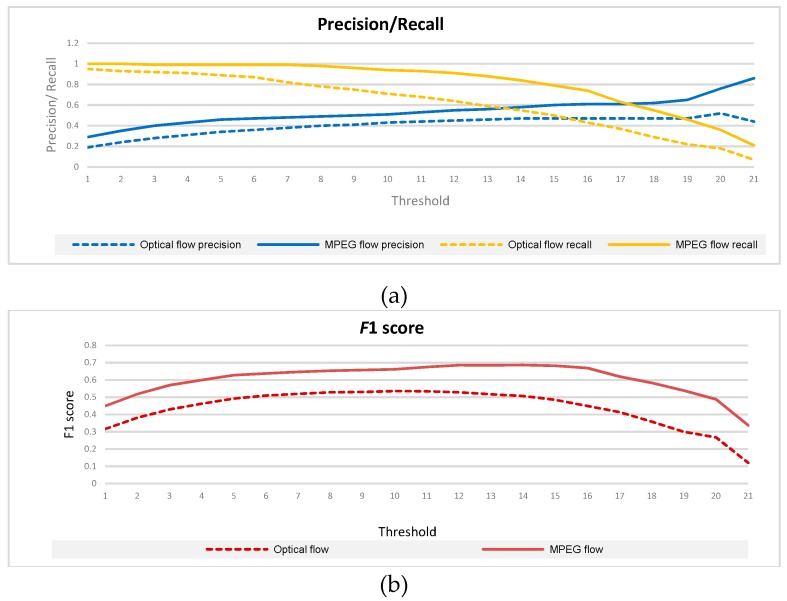
MPEG flow vs. Optical flow classifiers performance. (**a**) Precision/recall. for different threshold values. (**b**) *F*1 score.

The temporal filter percentile parameter was optimized using precision and recall values as a function of post processing filter threshold. To assess the usefulness of MPEG flow features we carried out experiments with two kinds of optic flow features: the MPEG flow and the Farneback optical flow [30] resized to our input format. Results for the first stage filter, using two different version of optical flow feature estimation are given in Figure 6. Solid curves correspond to MPEG flow, while dashed-dotted curves are obtained by using Farneback optical flow algorithm. Note that the prediction scores are obtained using frame level labeling. As depicted in the Figure 6 precision is increasing with the threshold θ, while the recall is decreasing.

(12) θ=p∗(2T+1)

The result shows that both precision and recall are higher for the MPEG flow for any threshold value, so we kept in further experiments only results for the MPEG flow. The variation of *F*1 score for MPEG flow features, as a function of threshold parameter *θ* is illustrated in Figure 6b.

**Figure 7:**
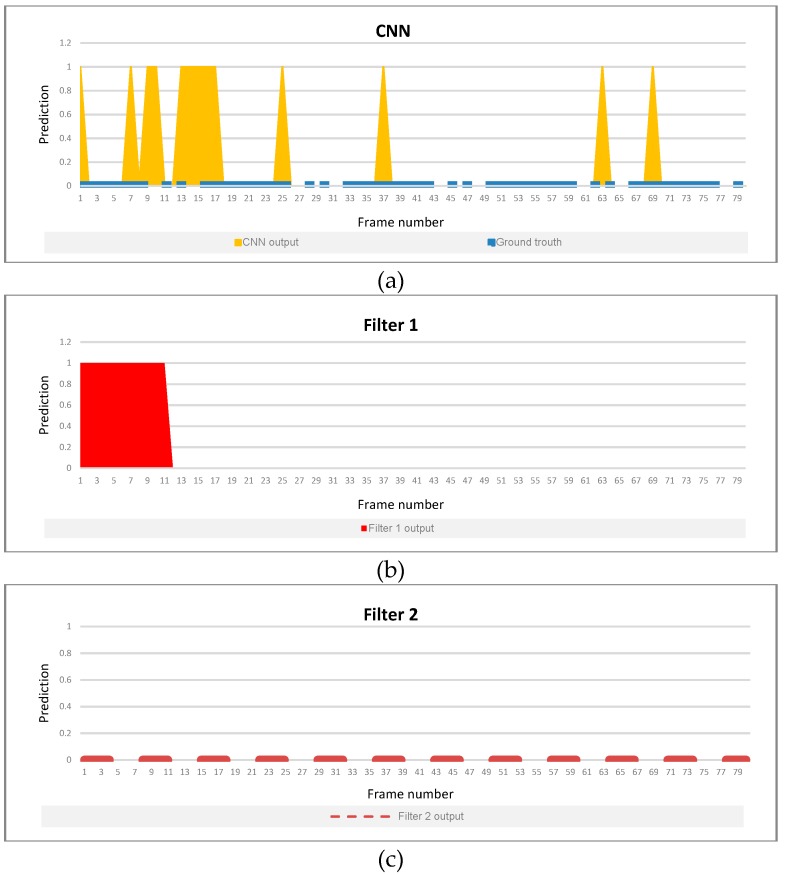
Output collected on different stages. (**a**) CNN prediction. (**b**) Filter 1 prediction. (**c**) Filter 2 prediction.

To maximize the F1 score for the first filter, we select the threshold *θ* = 14, which corresponds to the parameter *p* = 66%. However, in case of some non-violent sequences, the output of this filter is still influenced by noise introduced by very short dynamic movements happening in the scene. This case is depicted in Figure 7b, where the first filter fails to remove all the noise presented in the CNN output (Figure 7a). 

The second filter succeeds to solve the problem by considering a longer sequence, and by adding a new threshold which depends on the time window length (Figure 7c). The parameters of it have been designed in order to minimize the false positive rate, with the constraint of obtaining a recall of 100%. This optimization was carried out using labeling at video clip level. After the optimization process, the value of the parameter *θ* was 7, which corresponds to *p* = 100%.

## 5. Experiments

We use as a testbed for our experiments a Raspberry PI3 node integrating a Quad Core 1.2 GHz processor, 1 GB RAM, and a USB camera. The camera was set to 640 × 480 resolution at 25 fps with MPEG-4 encoding. The average time measured for one frame inference is 19.783 ms. During the inference, the solution uses less than 85 MB of RAM and about 48% CPU processing power. The implementation was made in C++ using the QtCreator 4.2.0 integrated development environment, the gcc 6.2.1 compiler, and the OpenCV 3.4.0 graphical library. The MPEG video stream from the camera was decoded using the ffmpeg 3.0 library. Decoding of a video frame takes less than 2 ms. For the training purpose, the DNN was re-implemented in Python using the Tensorflow 1.9 framework. In this step we used an Intel I5 PC equipped with a NVidia 1080Ti GPU as mentioned in the previous section.

Training and validation data have been obtained from two already mentioned databases [48,49] that contain 8765 classified frames. From these, 1322 frames correspond to violent behavior, and the rest are frames capturing normal behavior. Additionally, dataset augmentation technique has been applied to increase the variety.

To validate the performance of the proposed violent behavior detection method, we divided the video data into 86 clips, following the procedure adopted in [9]. All clips contain a type of activity from the following categories as attack, walk, run and group. All frames without motion detected were excluded since their classification is trivial. The class of violence was represented by attack clips, while the other activities are considered non-violent. A clip is classified as containing violence if at least one output of the second stage filter predicts this class.

In a first set of experiments we aim to optimize the recall. The results are synthesized by the confusion matrix in Table 1 and they correspond to a threshold value of 14 for the first filter, and a value of 7 for the second filter. We obtained 100% for the recall, 26.76% for the false positive rate, and 77.9% for the accuracy. Therefore, no violent event is missed and only 26.76% of the non-fight clips are predicted as false alarms. The aim of the second set of experiments was to optimize the accuracy by varying the first filter threshold. The best accuracy obtained was 86.93%, for a threshold value of 21. A comparison of the results obtained in the second set of experiments with other methods on the BEHAVE dataset is given in the Table 2. The ARENA dataset [49] contains only two video clips with violence. Our solution applied here recognize both, but we do not consider them in the reported results.

Zhang et al. [9] claim slightly higher accuracy on the BEHAVE dataset, 87.17%, but they do not directly report recall data, which is more important in our application. Another work [58], using the same dataset, reports results in the form of true positive versus false positive rate graph. From the graph, the false positive rate corresponding at the 100% while recall seems to be undistinguishable close to our result. 

The processing time was obtained for a 256 × 256 observation window applied on 1280 × 720 frames. In general, to deal with various video resolutions, every video frame should be split into sub windows of 256 × 256 pixels size.

We use a focus of attention approach by selecting only one window in each step. We start with the window which is the most active and discard windows with low activity. Activity was measured in our experiments by the sum of motion vector magnitudes in the window. From every window, a set of 16 × 16 motion vectors are extracted representing the motion features, fed to the CNN. Alternatively, it is possible to use arbitrarily large observation window. However, this implies a CNN reconfiguration and leads to a less efficient implementation.

For usability validation we use our setup in an outdoor lab environment on 10 predefined scenarios, five of them involving violence. The system operated on the 640 × 480 pixels 25 fps video stream and achieved an accuracy of 100% detection of violent sequences.

## 6. Conclusions

We propose in this work a DNN approach for sensor network violence detection, designed for urban area surveillance that provides automatic violent behavior detection. The novelty of the approach is represented by exclusively using, as input for the DNN, the motion features extracted from MPEG stream. We proved that using the features embedded in MPEG stream we avoid optical flow computation. Therefore, the solution is adapted to low resource distributed processing. We design a new architecture consisting of a cascade of a deep convolutional neural network and a time domain classifier. This allows separation of time domain and spatial processing. An advantage of relying exclusively on motion features consists of making the solution independent on illumination changes and on the color spectrum variations. We are confident that our system can easily accommodate also to night surveillance conditions including infrared vision. Using this architecture, we achieve state of the art performance, running on low computational embedded architecture with Raspberry PI sensor nodes. 

The performance tests were carried out on two standard datasets. This allows comparison with existing works but introduce some limitation of experiments. The datasets do not include a large variety of scales and camera angles therefore they do not allow extensive scalability tests. Retraining may be needed for applications that include such cases. 

As we targeted our algorithm to work with static cameras, it cannot handle camera motion. Another limitation relates with detection of crowd violence. Although the data sets used in our work include fights involving several persons, they do not contain crowd violence. We also do not detect explosions or any types of weapons. To cope with these, we propose to extend our method in the future with weapons recognition and therefore to cover also terrorist threats detection.

## Figures and Tables

**Table 1:** Confusion matrix for the best recall (100%).

		Labeled class	
		Violence	No violence
Predicted class	Violence	15	19
	No violence	0	52

**Table 2:** Detection results on the BEHAVE dataset.

Algorithm		ACC ± SD	AUC
Existing algorithms	HOG+BoW [32]	58.69 ± 0.35%	0.6322
	HOF+BoW [32]	59.91 ± 0.28%	0.5893
	HNF+BoW [32]	57.97 ± 0.31%	0.6089
	ViF [32]	82.02 ± 0.19%	0.8592
	MoSIFT+BoW [4]	62.02 ± 0.23%	0.6578
	RVD [59]	85.29 ± 0.16%	0.8878
	AMDN [42]	84.22 ± 0.17%	0.8562
	MoWLD+BoW [9]	83.19 ± 0.18%	0.8517
	MoWLD+SparseCoding [9]	85.75 ± 0.15%	0.8891
	MoWLD+KDE+SparseCoding [9]	87.17 ± 0.13%	0.8993
	Proposed method	86.93 ± 0.21%	0.9543
